# Supplementary material for: Proteasomal degradation of the tumour suppressor FBW7 requires branched ubiquitylation by TRIP12
Source: Nat Commun. 2021 Apr 6;12:2043. doi: 10.1038/s41467-021-22319-5 (PMC8024278; doi:10.1038/s41467-021-22319-5)
Supplement: Supplementary file 1 — Supplementary Information [file 41467_2021_22319_MOESM1_ESM.pdf]

Supplementary Figure 1

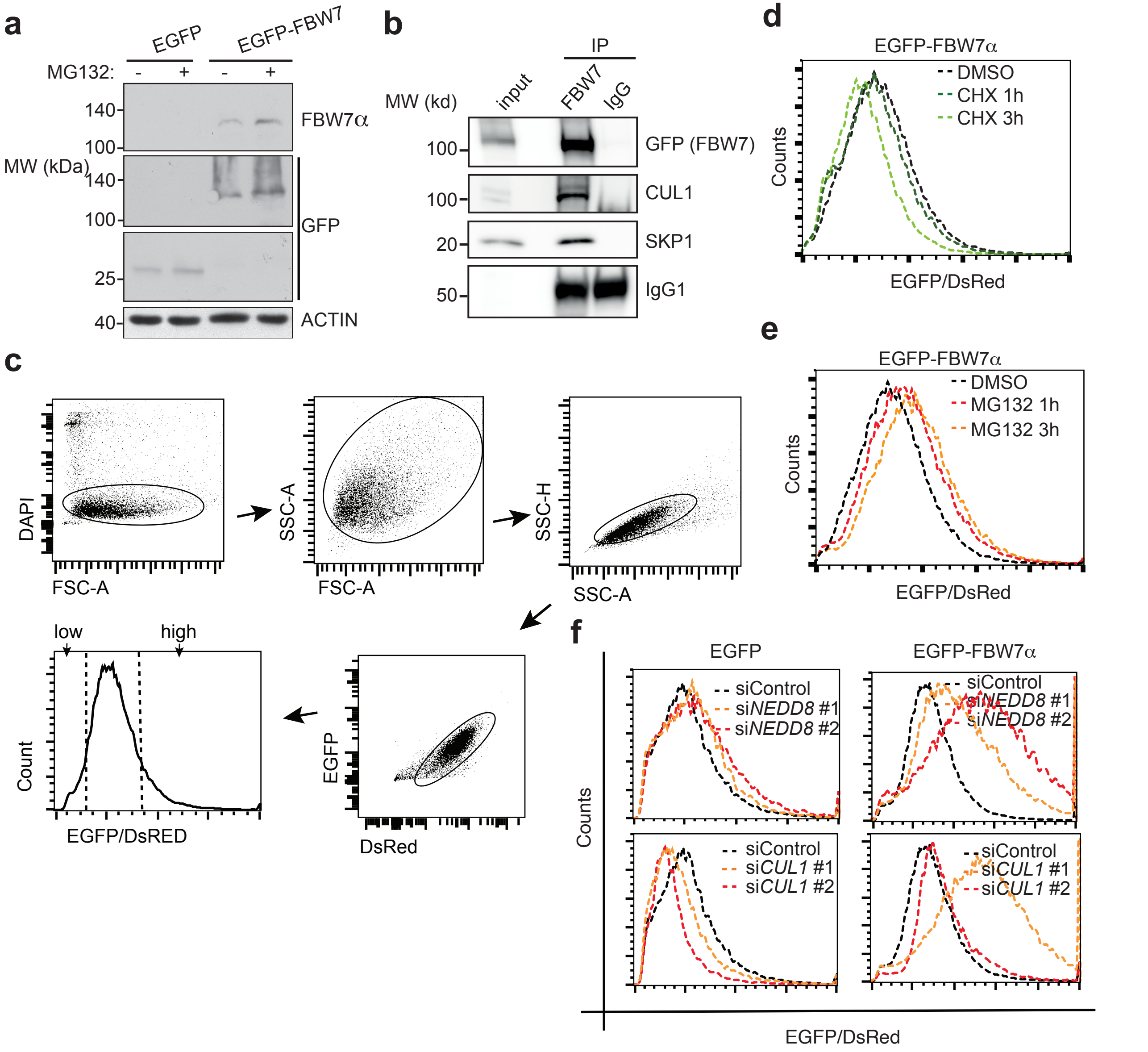

Supplementary Figure 1, related to Figure 1: Validation of FACS-based approach to assay FBW7 protein stability. (**a**) Western blot validation of DsRed-IRES-EGFP-FBW7α construct. (**b**) Co-IP/Western blot confirmation of EGFP-FBW7α interacting with its endogenous SKP1/CUL1 partners. (**c**) Gating strategy for FACS sorting and validation of selected hits. DAPI negative > SSC-A vs FSC-A > SSC-H vs SSC-A > EGFP+/RFP+ (double positive) > EGFP/DsRed cells were sorted in 2 bins based on a low or a high EGFP/DsRed ratio. Similar gating was done for validation of selected hits except sorting (**d**, **e**) FACS validation of DsRed-IRES-EGFP-FBW7α plasmid in the presence of a proteasome inhibitor (MG132) and a protein translation inhibitor (CHX). (**f**) FACS analysis validating CUL1 and NEDD8 as negative regulators of EGFP-FBW7α protein. Data are representative of at least 3 independent experiments.

Supplementary Figure 2

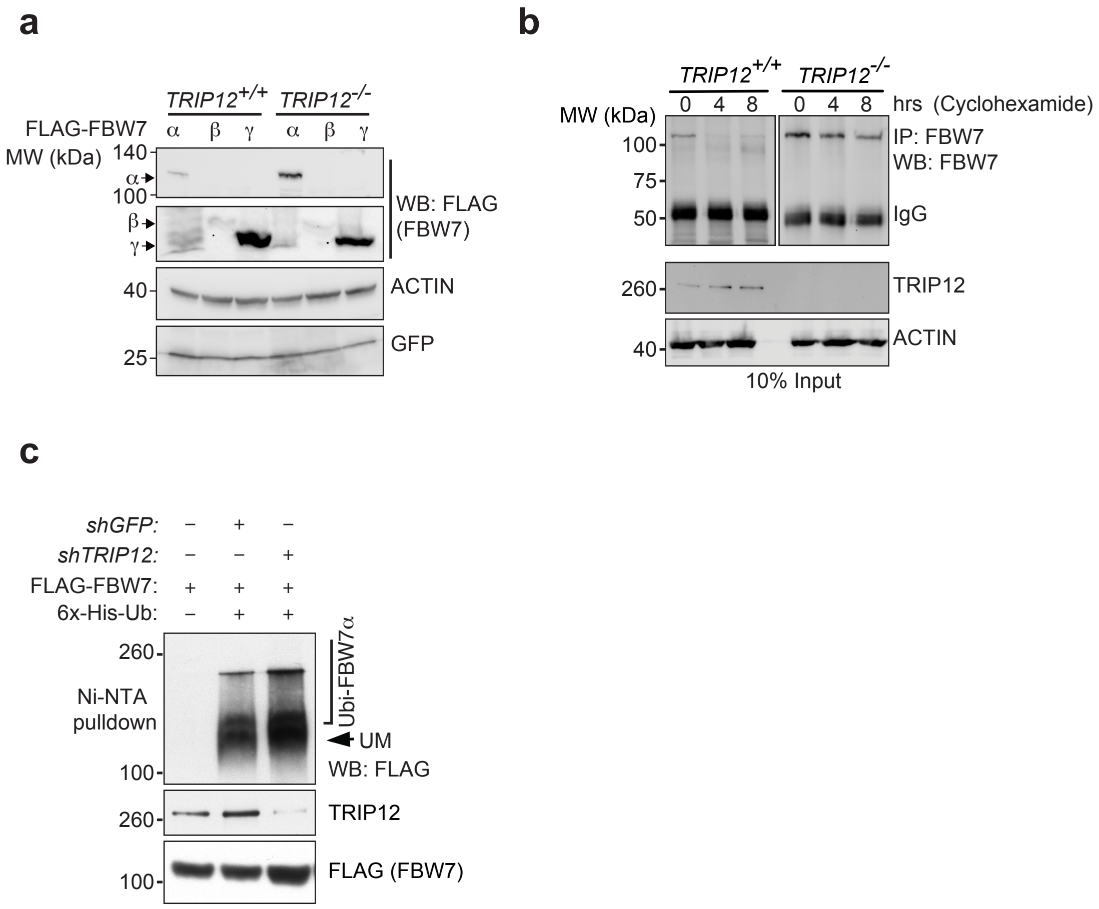

Supplementary Figure 2, related to Figure 2: Western blots for FBW7 isoforms, endogenous FBW7 stability and ubiquitylated FLAG-FBW7. (**a**) Western blots from lysates of HEK293T *TRIP12*$^{+/+}$ and *TRIP12*$^{-/-}$ cells transfected with the indicated FLAG-tagged FBW7 isoforms. GFP plasmid was used as transfection control and subsequently GFP and Actin blots were used as loading controls. **Note:** non-continuous gels spliced together due to low signal from highly labile FLAG-FBW7β. (**b**) Protein stability of endogenous-FBW7 in *TRIP12*$^{+/+}$ and *TRIP12*$^{-/-}$ cells as judged by western blots of immunoprecipitated FBW7. (**c**) Ni-NTA pulldowns of His-ubiquitylated FLAG-FBW7 in indicated shRNA treated cells. Blots are representative of atleast 3 independent experiments in all panels.

Supplementary Figure 3

a

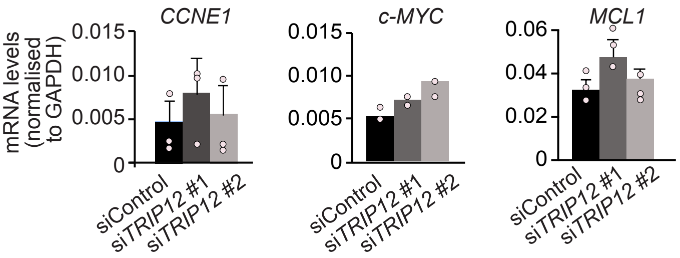

b

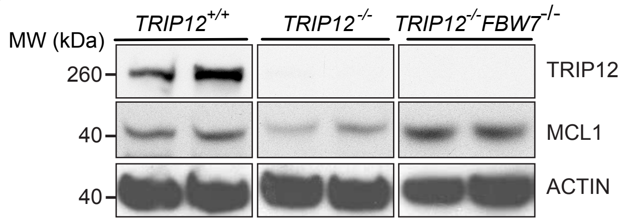

Supplementary Figure 3, related to Figure 3: qPCR of FBW7 indicated substrates and western blots for MCL1 in *TRIP12*-deficient cells. (**a**) mRNA expression data of the indicated genes in HEK293T cells incubated with the indicated siRNAs. Data represents Mean ± SD of n = 3 independent experiments except for *c-MYC* which was repeated twice with similar results. (**b**) Western blots from lysates of HCT116 cells with the indicated genotypes for TRIP12 and MCL1. Actin was used as loading control. Blots are representative of atleast 2 independent experiments performed in duplet for each genotype.

Supplementary Figure 4

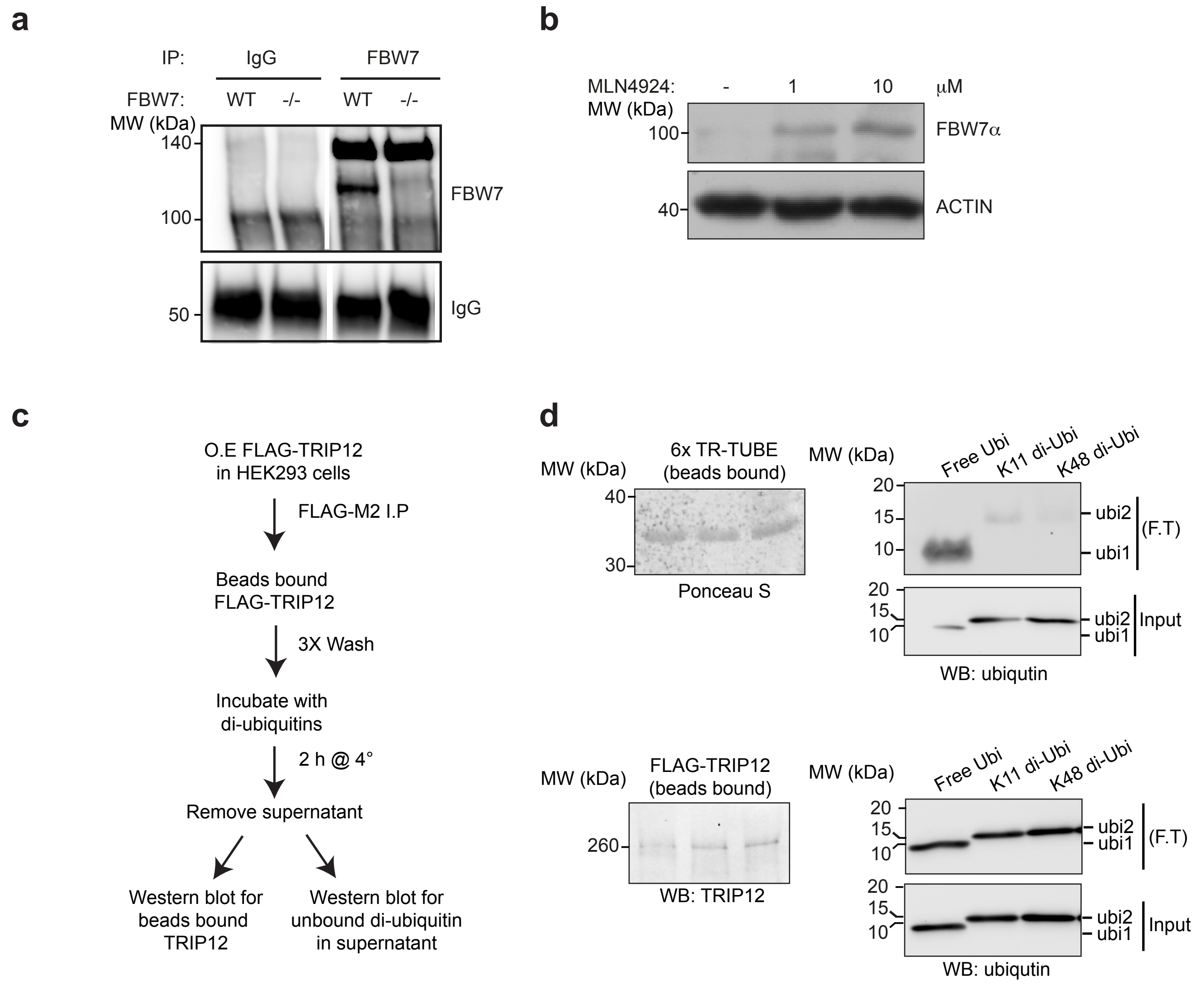

Supplementary Figure 4, related to Figure 4: Validation of endogenous FBW7 immunoprecipitation and lack of ubiquitin binding domain (UBD) in TRIP12. (**a**) Validation of endogenous FBW7 IP in *FBW7*$^{+/+}$ and *FBW7*$^{-/-}$ HCT116 cell lysates. Vertical white lines indicate splicing out of irrelevant lane in the middle of the blots. Samples were run on the same gel and processed in parallel. (**b**) Western blot confirmation of MLN4924 stabilizing FBW7α protein in HEK293T cells (**c**) Scheme for in vitro pulldown of diubiquitins of indicated linkages by beads bound full-length TRIP12, O.E = overexpression. (**d**) Western blots against indicated proteins showing lack of UBD in TRIP12, F.T (Flow through) compared to the positive control 6x-TR-TUBE which efficiently binds and remove diubiquitins from the supernatant. Blots are representative of 3 independent experiments.

Supplementary Figure 5

a

FBW7 lysines identified by mass spectrometry

Position	PEP	No. of GlyGly(K)	GlyGly(K) probabilities
171	3.5×10^{-13}	1	K(1)LDHGSEVR
186	5.2×10^{-15}	1	KPCKVSEYTSTTG LVPCSATPTTFGDLR
343	9.5×10^{-05}	1	VIK(1)PGFIHSPWK
404	3.5×10^{-04}	1	IVSGSDDNTLK(1)V WSAVTGK
412	6.3×10^{-03}	1	VWSAVTGK(1)CLR
609	8.6×10^{-05}	1	IWDIK(1)TGQCLQT LQGPNK
622	7.6×10^{-05}	1	TGQCLQTLQGPNK(1) HQSAVTCLQFNK

b

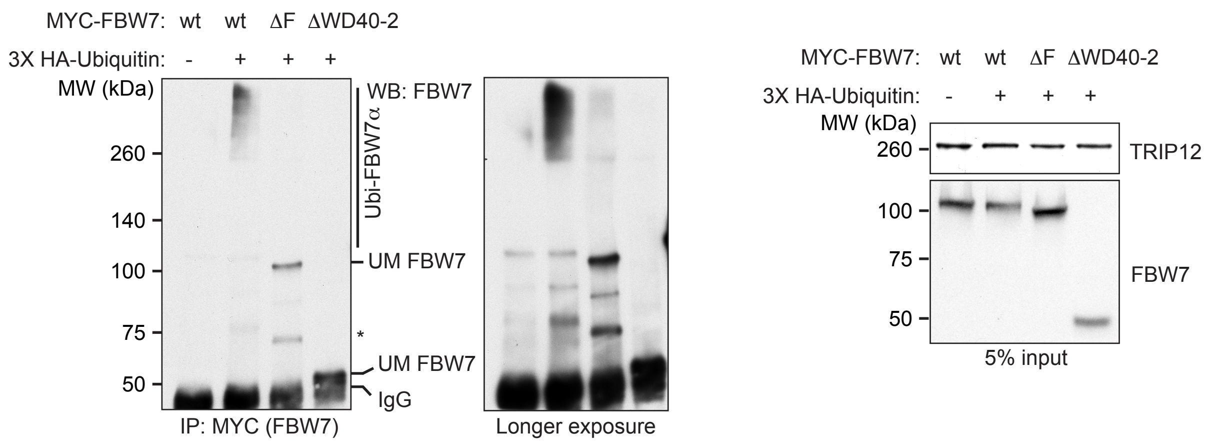

c **d**

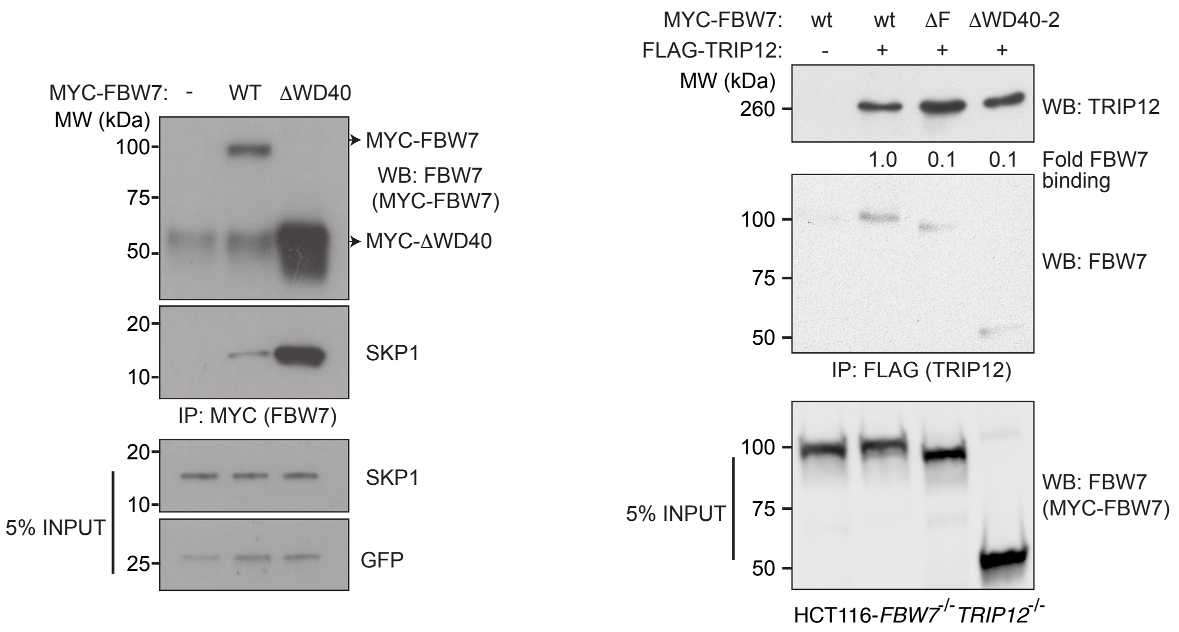

Supplementary Figure 5, related to Figure 5: Identification of ubiquitylated FBW7 lysines and TRIP12/FBW7 co-immuniprecitation. (**a**) List of FBW7α lysines and their respective peptides identified as ubiquitylated by mass spectrometry. (**b**) MYC pulldown of the indicated FBW7α wildtype or mutant proteins from HEK293T cells co-overexpressing 3x HA-ubiquitin and probed with FBW7α specific antibody. (**c**) Co-IP/Western blot confirmation of MYC-FBW7α wildtype and ΔWD40-2 mutant interacting with their endogenous SKP1 partner. (**d**) Western blots showing interaction of MYC-FBW7 wildtype, ΔFbox mutant, and ΔWD402 mutant with FLAG-TRIP12 in HCT116 cells of indicated genotype. Blots are representative of at least 3 independent experiments.

Supplementary Figure 6

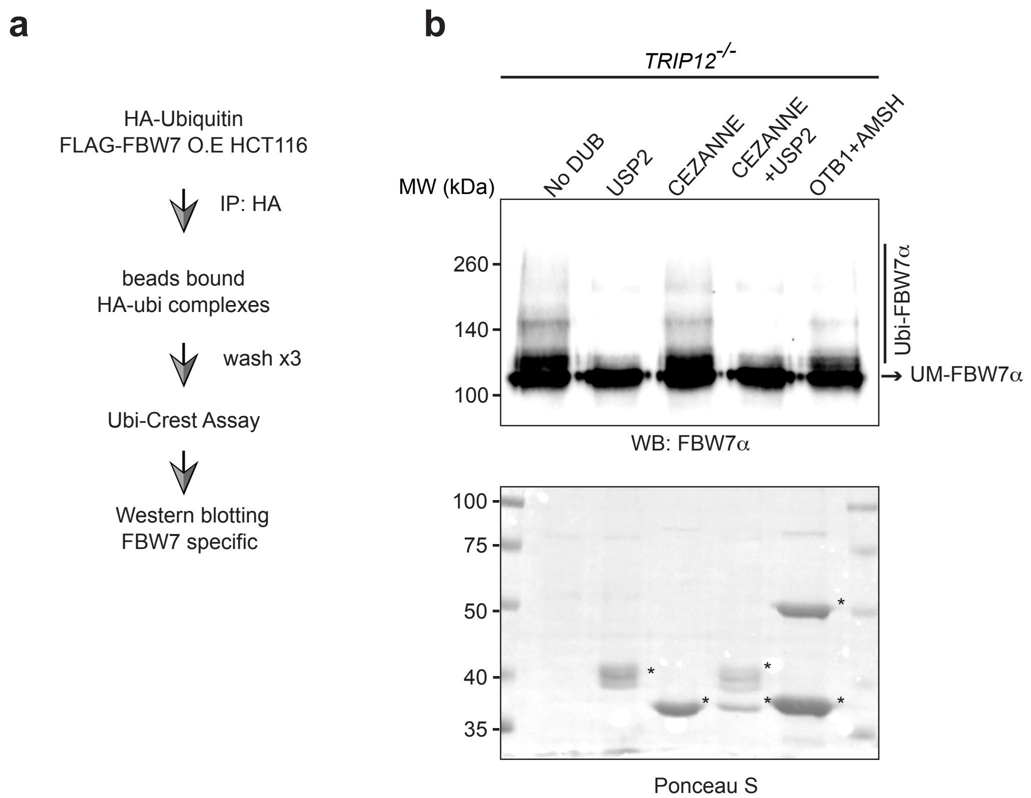

Supplementary Figure 6, related to Figure 6: Scheme and western blots for Ubi-Crest experiment on epitope-tagged FBW7. (**a**) Schematic for Ubi-Crest experiment in (b) on purified HA-ubiquitylated FLAG-FBW7 purified from *TRIP12*$^{-/-}$ HCT116 cells. (**b**) Western blot showing cleavage of beads-bound polyubiquitylated FLAG-FBW7 by the indicated DUBs, UM = unmodified. Blots are representative of at least 2 independent experiments.

Supplementary Figure 7

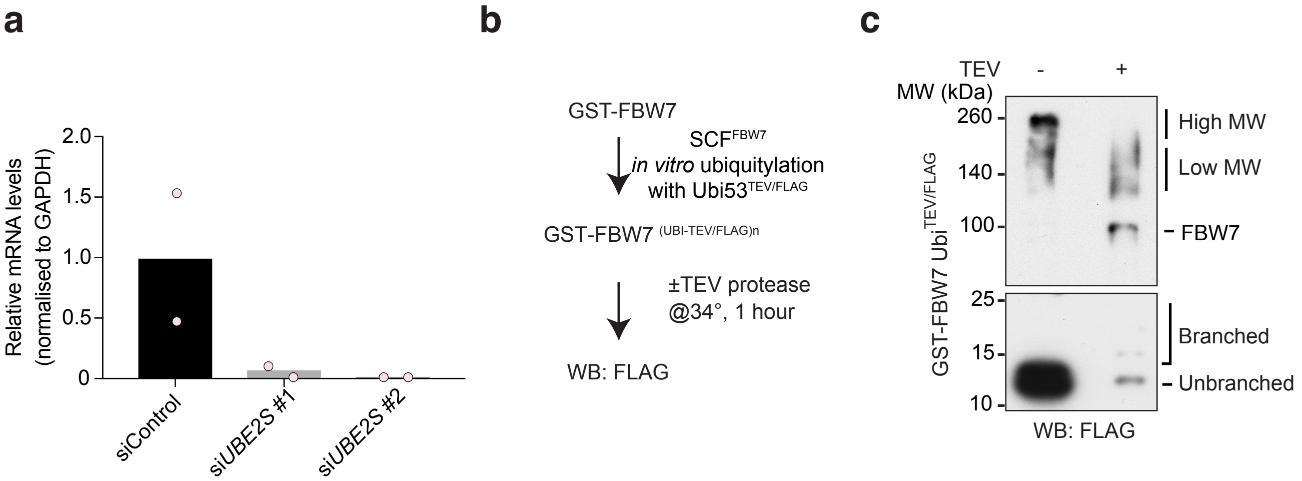

Supplementary Figure 7, related to Figure 7: Confirmation of si*UBE2S* by qPCR and control for Ubi53TEV/FLAG TEV-protease experiments. (**a**) qPCR data showing knockdown of *UBE2S* in HEK293T cells incubated with a control and two independent siRNAs targeting *UBE2S*. Data represents Mean of *n* = 2 independent experiments. (**b**) Scheme for in vitro ubiquitylation and TEV protease cleavage experiment in (c). (**c**) Western blot confirmation of TEV protease cleavage of high molecular weight polyubiTEV/FLAG-FBW7 conjugates generated in an autoubiquitylation reaction. Blot is representative of 2 independent experiments.

Supplementary Table 1: Primers used in the study

Name	Sequence	Purpose	Source
FBW7a_Attb1-Fwd	GGGGACAAGTTTGTACAAAAAAGCAGGCTTTAtgtgtgtcccgagaag	Gateway cloning of FBW7a	Sigma-Aldrich
FBW7a_Attb1-Rev	GGGGACCACTTTGTACAAGAAAGCTGGGTTCACTTCATGTCCACTC	Gateway cloning of FBW7a	Sigma-Aldrich
hUbi-Fwd3+ATTB1	GGGGACAAGTTTGTACAAAAAAGCAGGCTGTCAGATTTTCGTGAAAACCCT	Gateway cloning of ubiquitinTEV/Flag mutants	Sigma-Aldrich
hUbi-Rev3+ATTB2	GGGGACCACTTTGTACAAGAAAGCTGGGTTTTAACCACCACGAAGTCTCA	Gateway cloning of ubiquitinTEV/Flag mutants	Sigma-Aldrich
FBW7a_Attb1-Fwd	GGGGACAAGTTTGTACAAAAAAGCAGGCTTTAtgtgtgtcccgagaag	Gateway cloning of FBW7a deletion mutant	Sigma-Aldrich
FBW7a_WD40D1 Rev1	GGGGACCACTTTGTACAAGAAAGCTGGGTTtcatgttaacgtgtgaatgc	Gateway cloning of FBW7a deletion mutant	Sigma-Aldrich
FBW7a_Attb1-Fwd	GGGGACAAGTTTGTACAAAAAAGCAGGCTTTAtgtgtgtcccgagaag	Gateway cloning of FBW7a deletion mutant	Sigma-Aldrich
FBW7a_WD40D2 Rev1	GGGGACCACTTTGTACAAGAAAGCTGGGTTtcaatataaggtgtgtatac	Gateway cloning of FBW7a deletion mutant	Sigma-Aldrich
FBW7a_Attb1-Fwd	GGGGACAAGTTTGTACAAAAAAGCAGGCTTTAtgtgtgtcccgagaag	Gateway cloning of FBW7a deletion mutant	Sigma-Aldrich
FBW7a_WD40D3 Rev1	GGGGACCACTTTGTACAAGAAAGCTGGGTTtcaatgtcccactaatgttc	Gateway cloning of FBW7a deletion mutant	Sigma-Aldrich
FBW7a_Attb1-Fwd	GGGGACAAGTTTGTACAAAAAAGCAGGCTTTAtgtgtgtcccgagaag	Gateway cloning of FBW7a deletion mutant	Sigma-Aldrich
FBW7a_WD40D4 Rev1	GGGGACCACTTTGTACAAGAAAGCTGGGTTtcactcttctttgcatttct	Gateway cloning of FBW7a deletion mutant	Sigma-Aldrich
FBW7a K404R Fwd	gacaacactttaaGagtttggtcagc	Site directed mutagenesis	Sigma-Aldrich
FBW7a K404R Rev	gctgaccaaactCttaaagtgttgtc	Site directed mutagenesis	Sigma-Aldrich
FBW7a K412R Fwd	cagtcacaggcaGatgtctgagaac	Site directed mutagenesis	Sigma-Aldrich
FBW7a K412R Rev	gttctcagacatCtgcctgtgactg	Site directed mutagenesis	Sigma-Aldrich
FBXW7 K404,412R FWD	ctttaaGagtttggtcagcagtcacaggcaGatg	Site directed mutagenesis	Sigma-Aldrich
FBXW7 K404,412R REV	catCtgcctgtgactgctgaccaaactCttaaag	Site directed mutagenesis	Sigma-Aldrich
TRIP12 delta HECT Fwd	GGGGACAAGTTTGTACAAAAAAGCAGGCTTTATGTCCAACCGGCCTAAT	Gateway cloning of human TRIP12 deletion mutant	IDT
TRIP12 delta HECT Rev	GGGGACCACTTTGTACAAGAAAGCTGGGTTTCAGAACTGAGCAGCCAGAGA	Gateway cloning of human TRIP12 deletion mutant	IDT
TRIP12 delta WWE/HECT Fwd	GGGGACAAGTTTGTACAAAAAAGCAGGCTTTATGTCCAACCGGCCTAAT	Gateway cloning of human TRIP12 deletion mutant	IDT
TRIP12 delta WWE/HECT Rev	GGGGACCACTTTGTACAAGAAAGCTGGGTTTCATAATGCATTTCTTTGGGC	Gateway cloning of human TRIP12 deletion mutant	IDT
hUBE2S Fwd	CATCATCCGCCTGGTGTACAAG	qRTPCR	IDT
hUBE2S Rev	TAGGGTGGATCAGCAGGCAC	qRTPCR	IDT
GAPDH Fwd	TGAAGCAGGCATCTGAGGG	qRTPCR	Sigma-Aldrich
GAPDH Rev	CGAAGGTGGAAGAGTGGGAG	qRTPCR	Sigma-Aldrich
β-actin Fwd	GGATGCAGAAGGAGATCACTG	qRTPCR	Sigma-Aldrich
β-actin Rev	CGATCCACACGGAGTACTTG	qRTPCR	Sigma-Aldrich
c-MYC Fwd	CCTAGTGCTGCATGAGGAGA	qRTPCR	Sigma-Aldrich
c-MYC Rev	TCTTCCTCATCTTCTTGCTCTTC	qRTPCR	Sigma-Aldrich
Cyclin E Fwd	GCCAGCCTTGGGACAATAATG	qRTPCR	Sigma-Aldrich
Cyclin E Rev	AGTTTGGGTAAACCCGGTCAT	qRTPCR	Sigma-Aldrich
MCL1 Fwd	GTAATAACACCAGTACGGACGG	qRTPCR	Sigma-Aldrich
MCL1 Rev	TCCCGAAGGT ACCGAGAGAT	qRTPCR	Sigma-Aldrich

Supplementary Table 2: Oligos used in the study

Name	Catalogue number	Purpose	Source
si*COPS5* (*CSN5*)	SASI_Hs02_00342404	gene silencing	Sigma-Aldrich
	SASI_Hs01_00209042	gene silencing	Sigma-Aldrich
si*TRIP12*	SASI_Hs01_00130068	gene silencing	Sigma-Aldrich
	SASI_Hs02_00337169	gene silencing	Sigma-Aldrich
si*FBW7*	SASI_Hs01_00147263	gene silencing	Sigma-Aldrich
	SASI_Hs01_00147265	gene silencing	Sigma-Aldrich
si*CUL1*	SASI_Hs02_00335921	gene silencing	Sigma-Aldrich
	SASI_Hs01_00021744	gene silencing	Sigma-Aldrich
si*RBX1*	SASI_Hs01_00066597	gene silencing	Sigma-Aldrich
	SASI_Hs01_00066598	gene silencing	Sigma-Aldrich
si*NEDD8*	SASI_Hs01_00117479	gene silencing	Sigma-Aldrich
	SASI_Hs01_00117480	gene silencing	Sigma-Aldrich
si*UBE2S*	SASI_Hs01_00070712	gene silencing	Sigma-Aldrich
	SASI_Hs01_00070713	gene silencing	Sigma-Aldrich

Supplementary Table 3: crRNA or gRNA used in the study

gRNA	Sequence	Purpose	Catalogue No.	Source
Edit-R Human *TRIP12* crRNA 1	Available upon request from the vendor	Gene knockout using CRISPR/Cas9	CM-007182-01-0002	Dharmacon
Edit-R Human *TRIP12* crRNA 2	Available upon request from the vendor	Gene knockout using CRISPR/Cas9	CM-007182-02-0002	Dharmacon
Edit-R Human *TRIP12* crRNA 3	Available upon request from the vendor	Gene knockout using CRISPR/Cas9	CM-007182-03-0002	Dharmacon
T12_promoter_sgRNA3 Fwd	CACC GTAGGCATAAAAATAGGCCGA	CRISPR/SAM TRIP12 locus activation	Not applicable	Sigma-Aldrich
T12_promoter_sgRNA3 Rev	AAAC TCGGCCTATTTTTATGCCTAC	CRISPR/SAM TRIP12 locus activation	Not applicable	Sigma-Aldrich
T12_promoter_sgRNA4 Fwd	CACC CGCTCAAGTTGAGAATTTCA	CRISPR/SAM TRIP12 locus activation	Not applicable	Sigma-Aldrich
T12_promoter_sgRNA4 Rev	AAAC TGAAATTCTCAACTTGAGCG	CRISPR/SAM TRIP12 locus activation	Not applicable	Sigma-Aldrich
