# Supplementary material for: Proteasomal degradation of the tumour suppressor FBW7 requires branched ubiquitylation by TRIP12
Source: Nat Commun. 2021 Apr 6;12:2043. doi: 10.1038/s41467-021-22319-5 (PMC8024278; doi:10.1038/s41467-021-22319-5)
Supplement: Supplementary file 3 — Reporting Summary [file 41467_2021_22319_MOESM3_ESM.pdf]

nature research

Corresponding author(s): Axel Behrens
Omar Khan

Last updated by author(s): 23/02/2021

Reporting Summary

Nature Research wishes to improve the reproducibility of the work that we publish. This form provides structure for consistency and transparency in reporting. For further information on Nature Research policies, see Authors & Referees and the Editorial Policy Checklist.

Statistics

For all statistical analyses, confirm that the following items are present in the figure legend, table legend, main text, or Methods section.

n/a	Confirmed	
☐	☒	The exact sample size (n) for each experimental group/condition, given as a discrete number and unit of measurement
☐	☒	A statement on whether measurements were taken from distinct samples or whether the same sample was measured repeatedly
☐	☒	The statistical test(s) used AND whether they are one- or two-sided<br>*Only common tests should be described solely by name; describe more complex techniques in the Methods section.*
☒	☐	A description of all covariates tested
☒	☐	A description of any assumptions or corrections, such as tests of normality and adjustment for multiple comparisons
☒	☐	A full description of the statistical parameters including central tendency (e.g. means) or other basic estimates (e.g. regression coefficient) AND variation (e.g. standard deviation) or associated estimates of uncertainty (e.g. confidence intervals)
☒	☐	For null hypothesis testing, the test statistic (e.g. F, t, r) with confidence intervals, effect sizes, degrees of freedom and P value noted<br>*Give P values as exact values whenever suitable.*
☒	☐	For Bayesian analysis, information on the choice of priors and Markov chain Monte Carlo settings
☒	☐	For hierarchical and complex designs, identification of the appropriate level for tests and full reporting of outcomes
☒	☐	Estimates of effect sizes (e.g. Cohen's d, Pearson's r), indicating how they were calculated

Our web collection on statistics for biologists contains articles on many of the points above.

Software and code

Policy information about availability of computer code

Data collection	Maxquant (1.6.0.13), Samtools (0.1.16), BDFACS Diva 8.0.1
Data analysis	Microsoft excel 16.44, GraphPad Prism 8.0, and FlowJo 10.0

For manuscripts utilizing custom algorithms or software that are central to the research but not yet described in published literature, software must be made available to editors/reviewers. We strongly encourage code deposition in a community repository (e.g. GitHub). See the Nature Research guidelines for submitting code & software for further information.

Data

Policy information about availability of data

All manuscripts must include a data availability statement. This statement should provide the following information, where applicable:

- Accession codes, unique identifiers, or web links for publicly available datasets
- A list of figures that have associated raw data
- A description of any restrictions on data availability

All relevant data are included with the manuscript or available from the authors upon request.

Field-specific reporting

Please select the one below that is the best fit for your research. If you are not sure, read the appropriate sections before making your selection.

☒ Life sciences ☐ Behavioural & social sciences ☐ Ecological, evolutionary & environmental sciences

For a reference copy of the document with all sections, see nature.com/documents/nr-reporting-summary-flat.pdf

Life sciences study design

All studies must disclose on these points even when the disclosure is negative.

Sample size	No statistical tests were used to predetermine sample sizes. Experiments were usually repeated at least 3 times in triplicates. Sample sizes were chosen based on previous experience for such experiments in our lab (Ruiz et., JCI 2019). The level of significance was judged by respective t-tests.
Data exclusions	No data were excluded.
Replication	All experiments were repeated at least 3 times unless otherwise stated. Number of repeats are indicated in text or figure legends.
Randomization	Majority of the experiments were western blots because of which randomization was not possible.
Blinding	Blinding was not possible as western blots and cell viability experiments were performed by the same researchers. The shRNA library screen, deconvolution of shRNA, and pathway analysis were performed blindly. Mass spectrometry was also done by researchers blinded to the samples.

Reporting for specific materials, systems and methods

We require information from authors about some types of materials, experimental systems and methods used in many studies. Here, indicate whether each material, system or method listed is relevant to your study. If you are not sure if a list item applies to your research, read the appropriate section before selecting a response.

Materials & experimental systems

n/a	Involved in the study	
☐	☒	Antibodies
☐	☒	Eukaryotic cell lines
☒	☐	Palaeontology
☒	☐	Animals and other organisms
☒	☐	Human research participants
☒	☐	Clinical data

Methods

n/a	Involved in the study	
☒	☐	ChIP-seq
☐	☒	Flow cytometry
☒	☐	MRI-based neuroimaging

Antibodies

Antibodies used	Antibodies used for western blotting were anti-FBW7 (#A301-720A), and anti-TRIP12 (#301-814A) from Bethyl, anti-FLAG (#M2 clone), anti-HA HRP conjugate (#A190108P), anti-c-Myc Tag (#clone4A6) HRP conjugated, and anti-vinculin (#V9131) from Sigma, anti-GFP (#11814460001, Roche), anti-Actin HRP conjugated (#ab-49900), anti-GAPDH (#ab9485), anti-c-MYC-Y69 (#ab-32072), and anti-alpha-tubulin (#ab-7291) from Abcam, anti-CUL1 (#71-8700, Invitrogen), anti-p19Skp1 (#610530, BD Biosciences), anti-CyclinE (#sc-481), and anti-MCL1 (#54539) from Cell signaling, anti-conjugated ubiquitin (FK2) (#BML-PW8810, Enzo life sciences), and anti-Lys11 linkage specific (#MABS107), was all from Millipore.
Validation	All antibodies were commercially validated, previously published, or validated in the current study.

Eukaryotic cell lines

Policy information about cell lines

Cell line source(s)	Cell Services of The Francis Crick Institute for HEK293T, HEK293T-Cas9, HCT116, HCT116-Cas9, and HCT116+/+ and HCT116-FBW7-/- were from Bruce Clurman Lab (Fred Hutch, United States).
Authentication	All cells cells were authenticated using STR profiling and results checked against any available STR data for the parent line using commercial banks.
Mycoplasma contamination	All cell lines were obtained fresh mycoplasma free from Cell services of The Francis Crick Institute.
Commonly misidentified lines (See ICLAC register)	No commonly misidentified cell lines were used in this study.

Flow Cytometry

Plots

Confirm that:

☒ The axis labels state the marker and fluorochrome used (e.g. CD4-FITC).

☒ The axis scales are clearly visible. Include numbers along axes only for bottom left plot of group (a 'group' is an analysis of identical markers).

☒ All plots are contour plots with outliers or pseudocolor plots.

☒ A numerical value for number of cells or percentage (with statistics) is provided.

Methodology

Sample preparation	HEK293T-GPS-FBW7 cells were washed, trypsinized, counted, DAPI stained, and resuspended in sorting buffer (1% FBS in PBS). DAPI positive dead cells were excluded and double positive single scattered cells were sorted for low and high GFP/DsRed ratio. Mock vector transduced HEK293T-GPS-FBW7 cells were used as a control for gating purpose
Instrument	BD FACS Aria II and BD Fortessa A
Software	BD FACS Diva 8.0.1 and FlowJo 10.0.
Cell population abundance	For cell sorting, we used 1 billion cells/experiment and sorted 10 million live cells/population/replicate.
Gating strategy	DAPI negative live cells were selected, gated for scattered single cells by first gating for SSC-A/FSC-A and then for SSC-H/SSC-A or FSC-H/FSC-A. Single cells were then gated for GFP/DsRed double positive cells and GFP/DsRed ratio element was derived. Double positive cells were then sorted based on GFP/DsRed high or low ratio in to two distinct populations. Similar strategy was used for FACS validation of selected hits excluding the sorting.

☒ Tick this box to confirm that a figure exemplifying the gating strategy is provided in the Supplementary Information.
